# Difference in the foot intersegmental coordination pattern between female lacrosse players with and without a history of medial Tibial stress syndrome; a cross-sectional study

**DOI:** 10.1186/s13047-022-00513-y

**Published:** 2022-01-31

**Authors:** Hiroshi Akuzawa, Tomoki Oshikawa, Koji Nakamura, Ren Kubota, Norifumi Takaki, Naoto Matsunaga, Koji Kaneoka

**Affiliations:** 1 Faculty of Sport Sciences, Waseda University, 2-579-15, Mikajima, Tokorozawa, Saitama, Japan; 2 Graduate School of Sport Sciences, Waseda University, 2-579-15, Mikajima, Tokorozawa, Saitama, Japan; 3 General Education Core Curriculum Division, Seigakuin University, 1-1, Tosaki, Ageo, Saitama, Japan

**Keywords:** Shin splint, Modified vector coding technique, MTSS, Biomechanics

## Abstract

**Background:**

Medial tibial stress syndrome is a common sports related injury. Altered foot kinematics can be a risk factor for the injury. Since foot segments can move independently, intersegment coordination is important for proper foot function. This study aimed to compare the foot intersegmental coordination pattern and single segment kinematics between female lacrosse players with and without a history of medial tibial stress syndrome during drop jump.

**Methods:**

Twelve players with a medial tibial stress syndrome history and 12 players with no history were enrolled. Foot kinematics, including angle at landing and peak angle and excursion at the rearfoot, midfoot, and forefoot during single-leg drop jumps were analysed. Each segment motion data from landing to leaping was time-scaled to 100% to analyse the intersegmental coordination with a modified vector coding technique. Instant intersegmental coordination of every 1% was classified into four patterns (in-phase, two segments rotate in the same direction with similar amplitudes; anti-phase, two segments rotate in opposite directions; proximal phase, proximal segment dominantly rotates in the same direction compared to the distal segment; and distal phase, distal segment dominantly rotates in the same direction compared to the proximal segment). The percentage of intersegmental coordination pattern and kinematics in each segment were compared between the groups using the Student’s t test.

**Results:**

Groups with a history of medial stress syndrome showed a significantly higher percentage of proximal phase between the rearfoot and midfoot in the sagittal (Mean ± SD; history, 52.2 ± 17.9%, no history, 29.3 ± 16.7%; *p* = 0.004) and coronal planes (history, 40.3 ± 22.0%, no history, 15.9 ± 9.1%; *p* = 0.004). Dorsiflexion excursion (history, 34.5 ± 4.5°, no history, 29.6 ± 2.1°; *p* = 0.003) were significantly larger in a history of medial tibial stress syndrome group compared to no history group.

**Conclusions:**

Rearfoot dominant motion pattern relative to the midfoot may be related to medial tibial stress syndrome. Intersegmental coordination analysis may be useful for detecting abnormal foot coordination patterns. Also, stabilization for the rearfoot may be required rather than the midfoot for intervention.

## Background

Medial tibial stress syndrome (MTSS) is one of the most common running-related injuries in athletes. A relatively high incidence rate (13.6–20.0%) has been reported in athletic populations [1]. A previous study described that the mean recovery time to perform running training program was 105.2–117.6 days [2]. Because of this long recovery time, establishing an effective prevention program for MTSS is required. In order to establish a prevention program, risk factors for MTSS should be clarified. Several risk factors for MTSS have been described in much of the literature. Navicular drop is used to indicate midfoot pronation and medial longitudinal arch height [3], which was a common risk factor reported by several systematic reviews [4–6]. Another risk factor for MTSS is excessive and prolonged rearfoot pronation [7, 8]. It is speculated that excessive motions of the foot segments lead to higher eccentric contraction level of the soleus, flexor digitorum longus, and tibialis posterior muscles, which control arch height and rearfoot motion [9–12], and produces traction force induced longitudinal periostitis on the medial tibia [13–15].

However, navicular drop test only assesses the difference in distance between the floor and the lower border of the unloaded and loaded navicular bones in static postures. Hence this test does not reflect dynamic motion of the foot during weight bearing condition. Also, rearfoot motion is only single segment motion and it does not consider relative motion between foot segments. The foot consists of several segments, such as the rearfoot, midfoot, and forefoot. Each segment individually moves, and the coordination of these intersegmental motions is fundamental to adapt the foot’s shape to ground conditions. Because there are complex interactions between the segments during weight bearing activities, there is a limitation to detect abnormal motion pattern by assessing single segment motion. Accordingly, intersegmental coordination pattern analysis of the foot has been adopted [16–18]. The modified vector coding technique can represent coordinated motion patterns between the two adjacent segments [18]. Using this technique, instant intersegmental coordination pattern between the proximal and distal segments can be classified into one of the four patterns according to the rotation direction and amplitude: (1) in-phase, the proximal and distal segments rotate in the same direction with similar amplitudes; (2) anti-phase, the proximal and distal segments rotate in opposite directions; (3) proximal phase, the proximal segment dominantly rotates compared with the distal segment; and (4) distal phase, the distal segment dominantly rotates compared with the proximal segment [18].

To identify the specific intersegmental coordination pattern of the foot segments, which can be a risk factor for MTSS, sports related motion should be assessed. Single-leg drop jump is one of the tasks to be employed for assessment of the sports related injuries [19, 20]. Because single-leg drop jump requires landing shock attenuation and force transfer to leap up again immediately after landing, this motion is similar to the motion in sports.

This study aimed to clarify the foot intersegmental coordination pattern and each segmental angle differences between female lacrosse players with and without an MTSS history during drop jump task. It is hypothesized that the players with an MTSS history showed not only different single segment motion pattern, such as excessive eversion, but also altered intersegmental coordination patterns compared with the players without an MTSS history.

## Methods

### Study design

A cross-sectional study was conducted.

### Participants

All participants were recruited in a collegiate female lacrosse squad from July 2019 to September 2020. Self-report regarding history of injury was collected from 56 players in the squad. Following the self-report collection, a physical therapist who has a 17-year experience in the field of musculoskeletal and sports physical therapy interviewed all players and confirmed if they met the criteria or not. MTSS history is defined by the following criteria: 1) players experienced an atraumatic occurrence of pain and tenderness in the distal two-thirds of the medial tibia, which lasted at least one week; 2) pain was aggravated by running; and 3) training was limited by pain [19]. If players had ongoing symptoms during research period, these players were excluded. No history group was defined that players who had had absence of any pain or trauma in the lower limbs, which did not disrupt their training. Twelve players with a history of MTSS and 12 players without any history of leg injuries were recruited (Table 1). If players have an MTSS history in both lower limbs, with the more severe side was confirmed as the measured side. The physical therapist assessed the foot posture index 6 to rate the static foot posture in standing [21]. All the participants provided written informed consent prior to participation. The ethical committee of Waseda University approved this experiment (ID: 2019–082). This study was conducted in accordance with the Declaration of Helsinki.

**Table 1:** Participant characteristics

	MTSS history (*n* = 12)	No history (*n* = 12)	*P* value
Age, y^a^	19.9 ± 1.4	20.9 ± 1.2	0.05
Height, cm^a^	161.6 ± 4.2	160.5 ± 5.2	0.61
Weight, kg^a^	53.6 ± 3.4	55.7 ± 6.3	0.34
Measured Foot^b^ (Right/ Left)	8/ 4	8/ 4	
Foot Posture index 6^a^	1.3 ± 1.0	2.5 ± 2.4	0.15

^a^Values are mean ± SD, ^b^Values are n

### Three dimensional assessment

Three dimensional assessments were performed at the University’s laboratory. Twelve and four reflective markers were attached to the anatomical landmarks of the foot and shank, respectively, according to the Rizzoli Foot Model (RFM) [22]. Eight infrared cameras (Oqus; QUALIYSIS, Göteborg, Sweden) were set to collect marker trajectories on the foot and shank. Double-leg standing posture for 5 s was recorded to normalize the angles during the drop jump. Then, the players were instructed to perform a single-leg drop jump from a 30-cm box. Sufficient practice was allowed for the players to be familiar with the task before the measurement. Verbal instruction was provided to the players that they drop off the box and leap up vertically as high as possible immediately after landing. All the participants showed forefoot contact during drop jump. A force plate (Kistler, Winterthur, Switzerland) was set in front of the box to determine the landing and take-off timings. Three successful drop jumps were recorded at a sampling rate of 200 Hz [23]. A successful trial was defined when players landed again on the force plate, and all marker trajectories and force plate data were collected.

### Data processing

The segment angle data from the trials was synchronized to the force plate data. The ground reaction force data were filtered with a low-pass filter at 12 Hz [24]. A vertical component of the ground reaction force > 10 N was defined as the point of landing on the force plate [25]. The point at which the force became < 10 N after the landing was defined as the point of leaping. The segment angle data were analyzed using the Visual 3D software (C-motion Inc., Maryland, USA). Three-dimensional (3D) marker trajectories were filtered using a fourth-order Butterworth low-pass filter with a 6-Hz cut-off frequency [26]. The shank, rearfoot, midfoot, and forefoot segments were created according to the RFM [22]. Orientations of X-axis pointing forward, Y-axis pointing upward, and Z-axis pointing to the right according to a standardization proposal [27]. A Cardan sequence (Z-X-Y, representing dorsi-plantar flexion, eversion-inversion, and abduction-adduction) was used to calculate relative angles between segments. A positive value represents dorsiflexion, eversion, and abduction and a negative value means plantar flexion, inversion, and adduction. Three-dimensional rearfoot segment rotation relative to the shank was defined as rearfoot motion. Three-dimensional midfoot segment rotation relative to the rearfoot was defined as midfoot motion. Moreover, three-dimensional forefoot segment rotation relative to the midfoot was defined as forefoot motion. The segment angles of the rearfoot, midfoot, and forefoot were calculated. Since the segment motion in the transverse plane was relatively small, only the sagittal and coronal plane motions were calculated. The segment angles were normalized to the static double-leg standing posture. The segment angles at landing and peak angles from landing to leaping were reported. Segment excursion was measured as the difference between the angle at the landing and peak angle in the sagittal and coronal planes. Also, the segment angle data from landing to leaping in each trial were time-scaled to 100% for intersegmental coordination analysis.

### Repeatability assessment

Four players (one in the MTSS history group and three in the no history group) participated in the assessment for measurement repeatability. Repeated measurement was conducted more than a month after the first measurement. In order to assess the similarity of averaged kinematic waveforms of the foot segments acquired by two measurements, the coefficient of multiple correlation (CMC) was employed [28, 29]. The kinematic data from 4 participants were employed for repeatability analysis. The CMC value of 1 means perfect match and undefined value indicates dissimilar waveforms [29].

### Intersegmental coordination analysis

To calculate intersegmental coordination, the modified vector coding technique was utilized [17, 18]. A coupling angle (γ_i_) for each instant (i) during the normalized single-leg drop jump task was calculated by this technique to quantify intersegmental coordination according to Eqs. (1) and (2) [26].

1 $$ {\upgamma}_i= Atan\left(\frac{\uptheta_{D\left(i+1\right)}-{\uptheta}_{Di}}{\uptheta_{P\left(i+1\right)}-{\uptheta}_{pi}}\right)\frac{180}{\pi }\ \mathrm{if}\ {\uptheta}_{P\left(i+1\right)}-{\uptheta}_{pi}>0 $$

2 $$ {\upgamma}_i= Atan\left(\frac{\uptheta_{D\left(i+1\right)}-{\uptheta}_{Di}}{\uptheta_{P\left(i+1\right)}-{\uptheta}_{pi}}\right)\frac{180}{\pi }+180\ \mathrm{if}\ {\uptheta}_{P\left(i+1\right)}-{\uptheta}_{pi}<0 $$

Coupling angle (γ_i_) was calculated to show a value of 0–360° according to Eq. (3).

3 $$ {\upgamma}_i=\left\{\begin{array}{c}\ {\upgamma}_i+360\kern0.5em \mathrm{if}\ {\upgamma}_i<0\\ {}\ {\upgamma}_i\kern3.75em \mathrm{if}\ {\upgamma}_i\geqq 0\end{array}\right. $$

The mean coupling angle (γ_i_) of three trials was calculated using circular statistics of Eqs. (4) and (5).

4 $$ {\overline{x}}_i=\frac{1}{n}\sum \limits_{i=1}^n\cos {\upgamma}_i $$

5 $$ {\overline{y}}_i=\frac{1}{n}\sum \limits_{i=1}^n\sin {\gamma}_i $$

The following conditions (6) were applied to calculate the mean coupling angle (γ_i_) of 0–360°.

7 $$ {\overline{\gamma}}_i=\left\{\begin{array}{c}\kern0.5em Atan\left(\frac{{\overline{y}}_i}{{\overline{x}}_i}\right)\frac{180}{\uppi}\ \mathrm{if}\kern0.5em {x}_i>0,{y}_i>0\\ {} Atan\left(\frac{{\overline{y}}_i}{{\overline{x}}_i}\right)\frac{180}{\uppi}+180\ \mathrm{if}\kern0.5em {x}_i<0\\ {}\kern3.5em Atan\left(\frac{{\overline{y}}_i}{{\overline{x}}_i}\right)\frac{180}{\uppi}+360\ \mathrm{if}\kern0.5em {x}_i>0,{y}_i<0\end{array}\right. $$

Instant intersegmental coordination patterns were categorized into one of four patterns according to mean coupling angle (${\overline{\gamma}}_i$): (1) in-phase (22.5 ≦ ${\overline{\gamma}}_i$ < 67.5, 202.5 ≦ ${\overline{\gamma}}_i$ < 247.5); (2) anti-phase (112.5 ≦ ${\overline{\gamma}}_i$ < 157.5, 292.5 ≦ ${\overline{\gamma}}_i$ < 337.5); (3) proximal phase (0 ≦ ${\overline{\gamma}}_i$ < 22.5, 157.5 ≦ ${\overline{\gamma}}_i$ < 202.5, 337.5 ≦ ${\overline{\gamma}}_i$ ≦ 360); and (4) distal phase (67.5 ≦ ${\overline{\gamma}}_i$ < 122.5, 247.5 ≦ ${\overline{\gamma}}_i$ < 292.5) [18]. The percentage of each pattern in sagittal and coronal planes were calculated for each foot.

### Sample size calculation

Statistical power analysis was conducted with the G*power version 3.1 (Heinrich-Heine Universität, Germany). Because there was no previous data which compared intersegmental coordination pattern difference between athletes with and without MTSS history, results from this study were used for a priori power analysis. Minimum sample size was calculated with obtained effect size of 1.13, which was the minimum value among intersegmental coordination pattern comparison with significant difference, significance level of 0.05 and power level of 0.8. If sufficient number of participants was not achieved with the measured intersegmental coordination pattern data, further participant recruitment and measurements were planned.

### Statistical analysis

The SPSS Statistics version 26.0 (IBM, USA) was used for statistical comparison between groups. The Shapiro-Wilk and Levene tests were used to confirm the normal and equal data distributions, respectively. For group comparison in demographic data, foot posture index 6, peak angles, excursion, and percentage of each intersegmental pattern between the rearfoot and midfoot and the midfoot and forefoot in the sagittal and coronal planes, the Student’s t-test, Welch’s t-test, or Mann-Whitney U test were employed depending on the normal and equal distributions. The Cohen’s d was employed to calculate the effect size (ES) for group comparison (ES: small, 0.2–0.5; medium, 0.5–0.8; larger, more than 0.8) [30]. The α level was set at 0.05.

## Results

Statistical power analysis showed that 11 was the minimum sample size for each group. Therefore, further recruitment and measurement was not conducted. There were no significant differences in any demographic data and foot posture index 6 between groups. Repeatability analysis for kinematic waveform showed excellent repeatability for the sagittal plane motions (CMC: rearfoot, 0.97; midfoot, 0.98; forefoot; 0.91) and excellent to good repeatability for the coronal plane motions (CMC: rearfoot, 0.92; midfoot, 0.83; forefoot; 0.89).

**Fig. 1:**
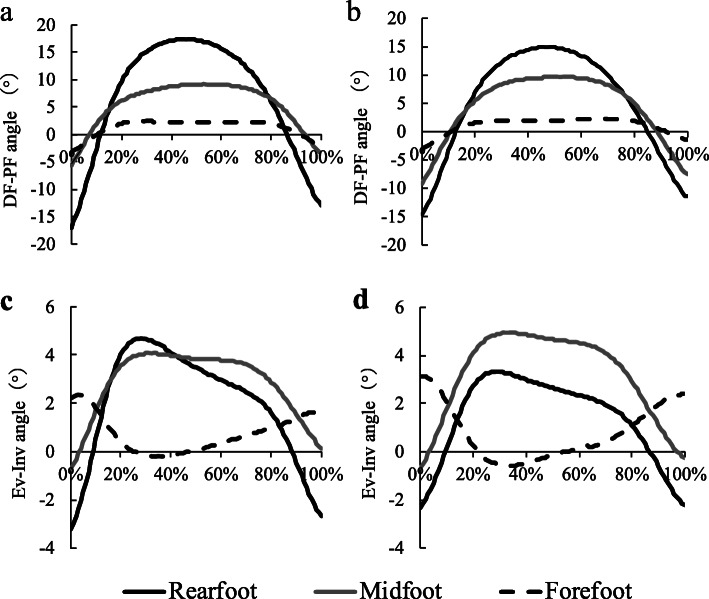
Averaged waveform of the rearfoot, midfoot, and forefoot segment angles in the MTSS history and no history groups. a: Dorsi-plantar flexion motion in the MTSS history group, b: Dorsi-plantar flexion motion in the no history group, c: Eversion/inversion motion in the MTSS group history, d: Eversion/inversion motion in the no history group. Positive value: dorsiflexion and eversion. Negative value: plantar flexion and inversion

**Fig. 2:**
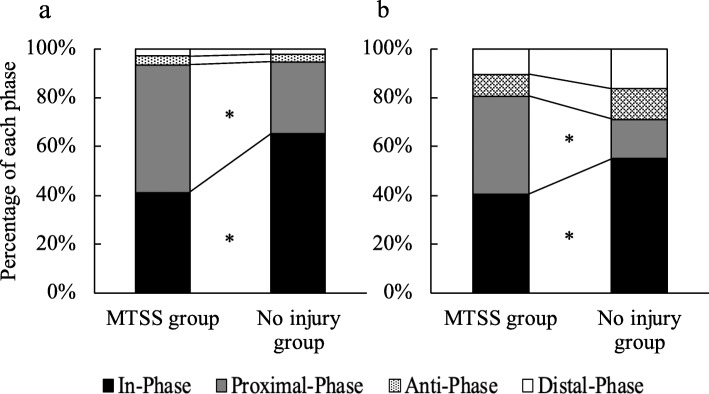
Percentage of each phase in drop jump. a: Dorsi-plantar flexion motion, b: Eversion/inversion motion. *: significant difference between groups

Figure 1 shows the averaged waveform of the rearfoot, midfoot, and forefoot segment angles from landing to leaping during drop jump task in the MTSS history and no history groups. In comparison of percentage of intersegmental coordination patterns between groups (Fig. 2 and Table 2), percentage of in-phase between the rearfoot and midfoot was significantly lower in the MTSS history group compared with the no history group in the sagittal (41.4 ± 16.9% vs. 65.4 ± 16.9%, *P* = 0.002, ES = 1.42) and coronal planes (40.5 ± 13.6% vs. 58.3 ± 17.5%, *P* = 0.011, ES = 1.13). Percentage of proximal phase between the rearfoot and midfoot was significantly higher in the MTSS history group compared with the no history group in the sagittal (52.2 ± 17.9% vs. 29.3 ± 16.7%, *P* = 0.004, ES = 1.32) and coronal planes (40.3 ± 22.0% vs. 15.9 ± 9.1%, *P* = 0.004, ES = 1.45). No significant differences were seen between groups for percentage of anti-phase and distal phase. Furthermore, there were no significant differences between groups for percentage of any phases between the midfoot and forefoot in the sagittal and coronal planes.

**Table 2:** Percentage of inter-segment coordination pattern comparisons between groups

Patterns	Inter-segments	Motions	MTSShistory^a^	Nohistory^a^	*P* value	95% CI	Effectsize
In-phase	Rear- mid	DF-PF	41.4 ± 16.9	65.4 ± 16.9	*0.002	− 38.3 to −9.7	1.42
		Ev-Inv	40.5 ± 13.6	58.3 ± 17.5	*0.011	− 31 to −4.5	1.13
	Mid- fore	DF-PF	28.9 ± 20.5	22.8 ± 18.5	0.453	−10.4 to 22.6	0.31
		Ev-Inv	13.9 ± 9.3	8.3 ± 5.5	0.086	−0.9 to 12	0.73
Anti-phase	Rear- mid	DF-PF	3.4 ± 2.6	3.1 ± 1.5	0.702	−1.5 to 2.1	0.16
		Ev-Inv	8.9 ± 6.9	9.8 ± 6.6	0.744	−6.7 to 4.8	0.14
	Mid- fore	DF-PF	9.6 ± 9.1	8.9 ± 6.4	0.837	− 6 to 7.3	0.09
		Ev-Inv	38.3 ± 19.2	50.9 ± 15.8	0.091	−27.5 to 2.2	0.72
Proximalphase	Rear- mid	DF-PF	52.2 ± 17.9	29.3 ± 16.7	*0.004	8.2 to 37.5	1.32
		Ev-Inv	40.3 ± 22.0	15.9 ± 9.1	*0.004	10.2 to 38.6	1.45
	Mid- fore	DF-PF	58.4 ± 18.5	63.1 ± 17.9	0.537	−20.1 to 10.7	0.26
		Ev-Inv	31.3 ± 22.8	29.3 ± 15	0.794	−14.3 to 18.4	0.11
Distalphase	Rear- mid	DF-PF	3.0 ± 2.3	2.1 ± 1.6	0.265	−0.7 to 2.6	0.47
		Ev-Inv	10.3 ± 11.4	15.8 ± 8.8	0.194	−14.2 to 3.1	0.55
	Mid- fore	DF-PF	3.1 ± 3.2	5.1 ± 4.8	0.24	−5.4 to 1.4	0.49
		Ev-Inv	16.5 ± 12	11.4 ± 5.6	0.199	−2.9 to 13	0.54

Abbrebiation: *DF-PF* Dorsiflexion- plantar flexion, *Ev-Inv*, Eversion- Inversion

^a^Valuses are mean ± SD, *Significant differences between groups

Regarding peak angles and excursions, group comparisons are shown in Table 3. The MTSS history group showed significantly larger peak angle (17.7 ± 2.4° vs. 15.0 ± 2.7°, *P* = 0.019, ES = 1.03) and excursion (34.5 ± 4.5° vs. 29.6 ± 2.1°, *P* = 0.003, ES = 1.38) of rearfoot dorsi-plantar flexion compared with the no history group. In contrast, the MTSS history group demonstrated significantly smaller midfoot dorsi-plantar flexion (15.4 ± 3.7° vs. 19.1 ± 3.8°, *P* = 0.024, ES = 0.99) and forefoot eversion/inversion excursion (2.8 ± 1.1° vs. 3.7 ± 1.0°, *P* = 0.011, ES = 1.13) than those in the no history group.

**Table 3:** Segment angle (in degree) comparisons between groups

	Segments	Motions	MTSShistory^a^	Nohistory^a^	*P* value	95% CI	Effectsize
Peak angle	Rearfoot	DF	17.7 ± 2.4	15.0 ± 2.7	*0.019	0.5 to 4.8	1.03
		Ev	5.1 ± 2.6	3.9 ± 4.1	0.393	−1.7 to 4.1	0.36
	Midfoot	DF	9.7 ± 2.9	9.9 ± 2.6	0.817	−2.6 to 2.1	0.1
		Ev	4.6 ± 1.8	5.4 ± 3.1	0.434	−3 to 1.3	0.33
	Forefoot	DF	2.8 ± 1.3	2.9 ± 1.1	0.836	−1.1 to 0.9	0.09
		Inv	0.6 ± 1	0.8 ± 1.2	0.775	− 0.8 to 1.1	0.12
Excursion	Rearfoot	DF-PF	34.5 ± 4.5	29.6 ± 2.1	*0.003	1.9 to 7.9	1.38
		Ev-Inv	8.3 ± 2.9	6.2 ± 2.1	0.059	− 0.1 to 4.2	0.81
	Midfoot	DF-PF	15.4 ± 3.7	19.1 ± 3.8	*0.024	−6.9 to − 0.5	0.99
		Ev-Inv	5.2 ± 2.4	6.1 ± 1.6	0.287	−2.7 to 0.8	0.45
	Forefoot	DF-PF	6.0 ± 2.4	5.9 ± 2.1	0.913	−1.8 to 2	0.05
		Ev-Inv	2.8 ± 1.1	3.9 ± 0.8	*0.011	0.3 to 1.9	1.13

Abbrebiation: *DF* dorsiflexion, *PF* plantar flexion, *Ev* eversion, *Inv* inversion

^a^Valuses are mean ± SD, *Significant differences between groups

## Discussion

This is the first study which identified the intersegmental coordination difference between athletes with and without MTSS history. As it was hypothesized that not only single segment motion but also intersegmental coordination showed significant difference between groups. The no history group showed that approximately 60% of intersegmental coordination patterns in the sagittal and coronal planes between the rearfoot and midfoot were in-phase, which the rearfoot and midfoot rotate toward same direction with similar amplitude [17]. However, segment motions dominantly occurred in the rearfoot compared with the midfoot in the MTSS history group (52% in sagittal plane and 40% in coronal plane).

There are two possible reasons which lead to the rearfoot dominant motion compared with the midfoot in the MTSS history group; the rearfoot motion was excessive or the midfoot motion was restricted. Excursions in sagittal plane were significantly higher in the rearfoot and lower in the midfoot in sagittal plane in the MTSS history group. This means that both excessive rearfoot and insufficient midfoot motions might be related to the rearfoot dominant motion in sagittal plane in the MTSS history group. These can be important findings because coronal plane alignment and motion abnormalities have been mainly reported as risk factor for MTSS by previous studies [4, 5, 8]. Because this study employed single-leg drop jump, which required larger ranges of motion in each segment compared to running or walking tasks, this may have highlighted different kinematic in the sagittal plane between the groups. The results differ from the hypothesis, the single segment angles in the coronal plane did not show any difference in the rearfoot and midfoot. This indicates that amount of motion in the rearfoot and midfoot in the coronal plane was similar; however, coordinated motion between the rearfoot and midfoot were different between groups. Taken together, not only instant single segment kinematic data, such as peak angle, but also coordination pattern in coronal plane throughout the task should be assessed to detect abnormal kinematics in MTSS patients.

Navicular drop have been considered as risk factors for MTSS and therapists often try to prevent from lowering navicular height by using insoles or taping for treatment and prevention [4–6, 31]. However, this study indicates that the rearfoot motion should be stabilized rather than the midfoot or the midfoot motion should be facilitated to correct coordination between segments. Insoles or taping may lead to too much restriction for the midfoot motion. A systematic review revealed that prior use of orthotics was one of risk factors for MTSS [4]. Use of insoles or taping may result in increased rearfoot motion as a compensation. Rearfoot motion is controlled by the calf muscles, including the gastrocnemius, soleus, tibialis posterior, tibialis anterior, flexor digitorum longus, and flexor hallucis longus [9–11]. One of the possible causes of MTSS is traction force induced longitudinal periostitis produced by the soleus, flexor digitorum longus, and tibialis posterior [13–15]. It is speculated that excessive motion of the rearfoot leads to higher eccentric contraction level of these muscles and produces higher stress on the medial tibia. A previous study showed that running for 30 min increased the tibialis posterior and flexor digitorum longus stiffness [32]. Also, runners with an MTSS history demonstrated higher tibialis posterior and flexor digitorum longus stiffness than that in no MTSS history [33]. Rearfoot dominant pattern may increase eccentric muscle contraction and traction force to the medial border of the tibia and related to MTSS. Thus, the midfoot support with insoles or taping should be carefully provided not to excessively restrict the midfoot motion. In addition, strengthening training for the extrinsic and intrinsic foot muscles, which have important function to control each foot segmental motion, may be important for dynamic intersegmental support [9–12, 34]. A previous study showed that there was a positive correlation between muscle activity level of the tibialis posterior and percentage of in-phase between rearfoot and midfoot in single-leg drop jump task [35].

There are some limitations in this study. Firstly, this study is a cross-sectional study which cannot identify MTSS risk factor. This study could only present feature of intersegmental coordination pattern of the foot for female lacrosse players with MTSS history. Secondly, since the participants in this study were only female lacrosse players, it is unclear whether same results can be seen in male athletes. Thirdly, because reflective markers were attached on the skin, skin motion artefact can influence the measurement reliability [36]. Even though repeatability of the measurements was good to excellent in this study, it should be carefully understood that these results did not present joint motions. Finally, this study measured barefoot motion because it is difficult to measure segmental foot motions with wearing a footwear. Care must be taken that footwear may influence on the foot kinematics. However, we believe that motion in barefoot most represents individual motion pattern.

## Conclusions

The MTSS history group showed significantly higher percentage of proximal phase between the rearfoot and midfoot in the sagittal and coronal plane motions. This indicates that the MTSS history group demonstrated rearfoot dominant motion pattern. There is a possibility that the rearfoot should be stabilized rather than the midfoot and intersegment coordination should be corrected for the treatment or prevention of MTSS. Further prospective study is required to detect whether intersegmental coordination difference can be an MTSS risk factor.

## Data Availability

The datasets used and/or analysed during the current study are available from the corresponding author on reasonable request.

## Abbreviations

MTSS: Medial tibial stress syndrome CMC Coefficient of multiple correlation
